# Supplementary material for: Iodine Intake and Related Cognitive Function Impairments in Elementary Schoolchildren
Source: Biology (Basel). 2022 Oct 14;11(10):1507. doi: 10.3390/biology11101507 (PMC9599038; doi:10.3390/biology11101507)
Supplement: Supplementary file 1 [file biology-11-01507-s001.zip › Supplementary Material S1.pdf]

Supplementary Material S1. Wechsler Intelligence Scale for Children – 3rd Edition (WISC-III; Portuguese version): Verbal-Performance IQs subtests characterization.

Subtests	*What is asked or done and what it means or measures.*
Verbal Subscales(VIQ)	
Information [a]	The child is given oral general information questions. Scoring is pass/fail. Measures fund of general knowledge; factual knowledge, long-term memory and recall.
Similarities	Items requiring child to describe how two given things are alike. Score on each item varies according to the degree to which the response describes a general property primarily pertinent to both items in the pair. Measures concrete, functional, and abstract concept formation.
Arithmetic [a, c]	This child is given oral, verbally framed math applications problems without paper or, for most problems, any visual aids at all. Scoring is pass/fail. Measures numerical reasoning, attention and short-term memory for meaningful information; attention, concentration and numerical reasoning.
Vocabulary	Words of increasing difficulty are presented orally and visually. Child required to define the words. Score (0-2) based on sophistication of definition. Measures verbal knowledge and concept formation.
Comprehension	Items that require child to explain what should be done in certain circumstances, the meaning of proverbs, why certain societal practices are followed, and so forth. The test measures practical judgment; common sense and the ability to understand and adapt to social customs. Score on each item varies (0-2 pts) according to the degree to which the response describes the most pertinent aspects of the question.
Digit Span [e]	The child is asked to repeat dictated series of digits forwards and other series backwards. The series begin with two digits and keep increasing in length, with two trials at each length. Measures short-term auditory memory for non-meaningful information and concentration.
Performance Subscales(PIQ)	
Picture Completion [a,c]	Several pictures, each having a part missing. Child must identify the missing part. Measures the ability to observe details and recognize specific features of the environment (i.e., whole to part discrimination). Also measures performance in deliberately focusing attention on a task.
Coding (A or B) [d]	Common shapes (Ages 6-7) or numbers 1-7 (ages 8 and older) are paired with symbols on a key presented to child. Child has 120 seconds to go through a grid of 90 numbers/shapes and place the correct symbol below each one. Measures visual-motor speed and complexity and motor coordination. There are two additional, optional extensions of the coding test that measure the child's skills in learning the coding process after completing the initial task.
Picture Arrangement [a, d]	The child is asked to sequence cartoon pictures to make sensible stories. Measures attention to visual detail; sequential reasoning; planning and social logical thinking knowledge.
Block Design [d]	This test measures the child's ability to look at the whole first, then break it into parts, and finally to reconstruct the whole. It provides blocks and pictures, and the child must put the blocks together to re-create what is in the picture of the blocks. Measures visual abstract ability; spatial analysis; abstract visual problem-solving.
Object Assembly [b, d]	The child is asked to assemble puzzles of cut-apart silhouette objects with no outline pieces. Measures part-whole reasoning; visual analysis and construction of objects.
Symbol Search [d, e]	The child is asked to decide if target symbols appear in a row of symbols and marking *Yes* or *No* accordingly. Measures speed and accuracy with which the child processes nonverbal information.
Mazes [b, c, e]	The child has to find the way out of a maze by using a pencil with no pencil lifting, points off for entering blind alleys. Measures graphomotor planning, visual-motor coordination and speed; fine motor coordination and planning and following directions.

[a] Supplemental in WISC-IV; [b] Subscale dropped in WISC-IV; [c] Time limit; [d] Time limit and bonuses for speed; [e] Additional subtests (optional). Adapted from Simões *et al.,* 2003.
