# Supplementary material for: Iodine Intake and Related Cognitive Function Impairments in Elementary Schoolchildren
Source: Biology (Basel). 2022 Oct 14;11(10):1507. doi: 10.3390/biology11101507 (PMC9599038; doi:10.3390/biology11101507)
Supplement: Supplementary file 1 [file biology-11-01507-s001.zip › Supplementary Material S2.pdf]

Supplementary Material S2. Raven´s Coloured Progressive Matrices (CPM) IQ scores (total and percentile scores).

**Iodine Status (n=256)**	***Total Score*** [1]	***Percentile Score*** [2]
**Severe Iodine Deficiency** (UIC <20 µg/L)	30.33 ± 5.590	79.89 ± 35.297
**Moderate Iodine Deficiency** (UIC 20-49 µg/L)	26.71 ± 5.136	51.57 ± 32.755
**Mild Iodine Deficiency** (UIC 50-99 µg/L)	26.14 ± 6.247	51.58 ± 32.770
**Adequate Iodine Intake** (UIC 100-199 µg/L)	27.84 ± 4.973	54.51 ± 33.339
**More than Adequate Iodine Intake** (UIC 200-299 µg/L)	29.33 ± 2.887	56.67 ± 24.664
	p=0.117[3]	p=0.384[3]

[1] Mean ± SD (total score 1(min)-36(max) points);

[2] Mean ± SD;

[3] Kruskal-Wallis test.
