# Supplementary material for: Iodine Intake and Related Cognitive Function Impairments in Elementary Schoolchildren
Source: Biology (Basel). 2022 Oct 14;11(10):1507. doi: 10.3390/biology11101507 (PMC9599038; doi:10.3390/biology11101507)
Supplement: Supplementary file 1 [file biology-11-01507-s001.zip › Supplementary Material S3.pdf]

Supplementary Material S3. Raven´s Coloured Progressive Matrices (CPM) (Total and percentile scores) for Moderate Iodine Deficiency and Adequate Iodine Intake subgroups.

Iodine Status	Measure (CPM)		
	Total Score [1]	***Percentile Score*** [2]	***IQ Level***
**Moderate Iodine Deficiency** (UIC 20-49 µg/L, n=30)	25.63 ± 5.537	45.47 ± 33.961	IV – Bellow average [3]
**Adequate Iodine Intake** (UIC 100-199 µg/L, n=30)	28.37 ± 5.255	60.43 ± 33.109	III - Average [4]
	p=0.055[5]	p=0.089[5]	

[1] Mean ± SD (total score 1(min)-36(max) points);

[2] Mean ± SD;

[3] Percentile ≤25;

[4] Percentile 25-75

[5] t-test.
